# A 28-GHz Switched-Beam Antenna with Integrated Butler Matrix and Switch for 5G Applications

**DOI:** 10.3390/s21155128

**Published:** 2021-07-29

**Authors:** Sujae Lee, Yongho Lee, Hyunchol Shin

**Affiliations:** Department of Electronics Convergence Engineering, Kwangwoon University, Seoul 01897, Korea; sori9505@kw.ac.kr (S.L.); dldyd91@kw.ac.kr (Y.L.)

**Keywords:** beamforming, switched-beam, Butler matrix, branch line coupler, switch, microstrip antenna, millimeter wave, 5G

## Abstract

This work presents a 28-GHz Butler matrix based switched-beam antenna for fifth-generation (5G) wireless applications. It integrates a 1 × 4 microstrip antenna, a 4 × 4 Butler matrix, and a single-pole four-throw (SP4T) absorptive switch in a single planar printed circuit board and is housed in a metal enclosure. Co-integration of a packaged switch chip with the Butler matrix based switched-beam antenna greatly enhances the form factor and integration level of the entire system. A wideband two-section branch line coupler is employed to minimize the phase and magnitude errors and variations of the Butler matrix. The aluminum metal enclosure stabilizes the electrical performances, reduces the sidelobes, and improves the structural stability. The fabricated antenna with the metal enclosure assembled has a dimension of 37 × 50 × 6.2 mm^3^. With an RF input signal fed to the antenna’s input port through a single Ka-band connector, and the switching states chosen by 2-bit dc control voltages, the antenna successfully demonstrates four directional switched beams. The beam switching operations are verified through the over-the-air far-field measurements. The measured results show that the four beam steering directions are −43°, −17°, +10°, +34° with side lobe levels < −5.3 dB at 28 GHz. The antenna also shows reasonably wideband radiation patterns over 27–29 GHz band. The 10-dB impedance bandwidth is 25.4–27.6 GHz, while a slightly relaxed 8-dB bandwidth is 25.2–29.6 GHz. Compared to previous works, this four-directional switched-beam antenna successfully exhibits the advantages of high integration level and satisfactory performances for the 28-GHz 5G wireless applications.

## 1. Introduction

The millimeter-wave (mm-wave) fifth-generation (5G) wireless communication demands high-gain directive antenna systems with a beam steering capability. It is needed to overcome significant loss caused by free-space propagation, building penetration, blockage, shadowing, and so on.

Two methods for the electronic beam-steering are possible: continuous and discrete methods [1]. The continuous beam-steering is realized by employing active beamforming circuits, by which the phase and magnitude at each antenna element are tuned so that its resulting beam direction is controlled in a continuous manner. On the other hand, the discrete beam-steering only allows for a predefined set of phase and magnitude at each antenna element, thus its resulting beam direction is set to one of the predefined finite set of angles. For the discrete beam-steering, a passive beamforming circuit is preferred to an active circuit because it is usually more advantageous in terms of hardware complexity, power consumption, and material cost.

The passive beamforming circuit is typically based on phase-shifting passive circuit networks such as Butler, Blass, and Nolen matrices [2]. Noting that a directional coupler is a key building component for the three matrices, Butler matrix requires a smaller number of couplers than Blass and Nolen matrices. For example, 4 × 4 Butler matrix requires 5 couplers, whereas Blass and Nolen matrices require 16. In addition, Butler matrix requires only half of terminating resistive loads compared to the others. These advantages make the Butler matrix the most favorable network for the passive switched-beam antenna.

Various mm-wave switched-beam antenna systems based on the Butler matrix have been reported previously, including [3,4,5,6,7,8,9,10,11,12] in Ka-band and [13,14,15] in V-band. All of them carefully investigated co-integrated designs and implementations of an array antenna and Butler matrix. Interestingly, the co-integration would not have been such a critical issue in a much lower few-GHz band as can be confirmed in [16,17,18]. It is because in the conventional non-mm-wave band, a simple modular design and connectorized assembly of the building components could be more straightforward and less expensive as the interconnections and integrations of the building components are not so much problematic as in the mm-wave band. The mm-wave band design should critically include co-integrated design and implementation processes with full considerations on the inter-connection and inter-coupling issues among the building components. However, none of the previous works [3,4,5,6,7,8,9,10,11,12,13,14,15] had successfully shown the co-integration of a switch in their antennas. Without incorporating a switch, they only could characterize their antennas by exciting one port of N × N Butler matrix, while terminating other ports with 50-Ω matching resistors [10]. If a switch was additionally integrated in their prototypes, the previous antennas [3,4,5,6,7,8,9,10,11,12,13,14,15] would possibly encounter drastic and sometimes severe performance degradations.

For integrating a switch in a planar printed circuit board (PCB), interfaces and transitions between the switch and Butler matrix after chip-mounting on PCB must be carefully designed. From the switch-integration viewpoint, previous reports by Fang et al. [19] and Kim et al. [20] were interesting because they successfully demonstrated a complete integration of switch, Butler matrix, and antenna array, but only in 1–2 GHz non-mm-wave band. In contrast, fully integrated switched-beam antenna systems in mm-wave band are not found much in literature. It is because the integration design process is more complicated and relevant mm-wave switch parts are not readily available. Malmqvist et al. [21] demonstrated a 24-GHz switched-beam antenna system adopting a micro-electromechanical system (MEMS) switch. However, the special fabrication process and excessively high driving voltage for the MEMS switch made the approach less attractive. Choi et al. [22] and Kuo et al. [23] adopted proprietary-designed foundry-processed CMOS switch integrated circuits (IC) in their 60-GHz switched-beam antennas. However, the lack of commercial availability of the switch would hinder its adoption in a further general form. Patterson et al. [24] demonstrated a 60-GHz switched-beam antenna system by integrating a commercially available GaAs PIN diode switch (part number HMC SDD-112 [25]). Yet, all the switches used in [22,23,24] were only used in a bare die form, not in a packaged form. We believe that those limitations make the previous works [22,23,24] still not attractive from the viewpoints of robust and stable high-volume fabrication. 

In this work, a switched-beam antenna is described for 28-GHz mm-wave 5G applications. Compared to the prior works, this work advances the integral design and fabrication technology by fully integrating the microstrip array antenna, Butler matrix, and packaged switch IC in a single planar PCB.

## 2. Design

**Figure 1:**
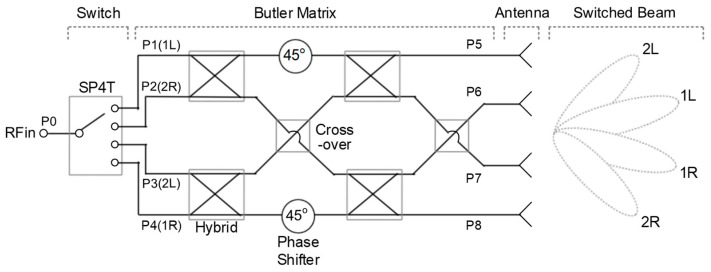
Switched-beam antenna system architecture.

Figure 1 shows the architecture of the switched-beam antenna system. It comprises a single-pole four-throw (SP4T) switch, 4 × 4 Butler matrix, 1 × 4 array antenna. The Butler matrix has four input ports P1–P4 and four output ports P5–P8. It comprises four hybrid couplers, two cross-overs, and two 45° phase shifters. A mm-wave RF input signal is fed to the RF input port P0. The SP4T switch selects a single port out of the four ports P1–P4, while the rest of the three ports are terminated to the 50-Ω matched resistance. This characteristic of the switch is referred to as non-reflective or absorptive. Depending on the selected signal-feeding port out of P1–P4, the four output signals from P5 through P8 have progressive phase values with a constant step of +45°, −135°, +135°, and −45°, respectively. Since two 3-dB hybrid couplers are involved in the input-to-output signal path in the Butler matrix, the insertion loss of an ideal Butler matrix is 6 dB unless any additional loss is considered. Thus, the output power at the four output ports P5–P8 will be nominally 6-dB lower than the input power at P0. Finally, four switched beam patterns denoted by 2L, 1L, 1R, 2R are produced according to the selected switch states of P3, P1, P4, P2, respectively. The ideal beam-direction angles are −45°, −15°, +15°, and +45° for 2L, 1L, 1R, and 2R, respectively.

The PCB stack-up consists of two-layer substrates and an intermediate prepreg layer for attaching the two. The two-layer substrates are identical having a dielectric constant ε_r_ of 3.0 and thickness h of 0.25 mm. The prepreg has a thickness of 0.1 mm. Since both sides of each layer are coated by a metal layer, four metal layers are formed in total. Among them, the outer two layers are used for the antenna and circuit realization, and the inner two layers are used only for ground planes for the microstrip-line based structures at the outer layers.

**Figure 2:**
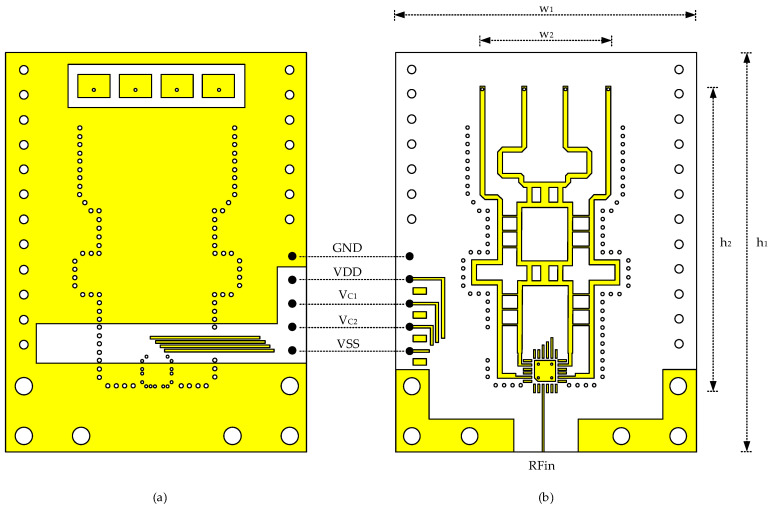
PCB layout. (**a**) antenna side (front), (**b**) beamforming circuit side (back).

Figure 2 shows the PCB layout. The front side used for the array antenna is shown in Figure 2a, and the back side used for the beamforming circuit is shown in Figure 2b. It has a total dimension of w_1_ = 36 mm and h_1_ = 48.1 mm. The front side is mostly covered by the ground plane to minimize unwanted EM couplings, while only limited regions are exposed for the array antenna and dc routings. Unlike the front side, the back side is not covered by the ground plane so that all the interconnections are designed in a microstrip line structure. Via fences are formed around the Butler matrix to improve the RF characteristics variability.

As shown in Figure 2a, the 1 × 4 microstrip patch array antenna is surrounded by the ground plane with a gap spacing of 1.2 mm. Each antenna element has a dimension of 3.8 × 2.82 mm^2^, and the element-to-element spacing and pitch are 1.2 and 5 mm, respectively. The microstrip antenna is designed first by following the theoretical expressions such as given in [26], and then performing optimizations through full three-dimensional (3D) electromagnetic (EM) simulations. For each element, an inductive probe feeding is adopted by using a through-hole via. The via diameter is 0.3 mm with an anti-pad diameter of 0.5 mm. Minimum via dimensions allowed by drilling process are used to alleviate unwanted adverse effects on the antenna’s radiation pattern.

The four antenna vias from the front side are connected to the back-side Butler matrix as shown in Figure 2b. The core dimension of the Butler matrix is w_2_ = 17.6 mm and h_2_ = 31.3 mm. The Butler matrix comprises four 90° hybrid branch-line coupler, two cross-overs, two 45° phase shifters. The interconnecting lines to the antenna at the upper side as well as the interconnecting lines from the switch at the lower side are carefully designed to minimize the phase and magnitude errors of the Butler matrix. All circuits are laid out in perfect symmetry. The SP4T switch is placed at the lower side as its footprint is shown in Figure 2b. The four output pins are connected to the upper Butler matrix, and the single RF input signal is connected to the RF connector at the PCB bottom side. 

A commercially available off-the-shelf part ADRF5045 [27] is employed for the SP4T switch. At the time of writing this paper, many switch ICs that can operate in S, C, X, and Ku bands are commercially available, but unfortunately not many in the desired 28-GHz Ka-band. This is why most previous antenna systems have presented only partially integrated prototype results without the switch integration [3,4,5,6,7,8,9,10,11,12,13,14,15], or employed only in-house proprietary switch circuits [21,22,23], even if they had shown fully integrated results. To the best of authors’ knowledge, this work is the first to present a fully integrated 28-GHz switched-beam antenna with a co-integration of a commercially available packaged switch chip.

The switch is absorptive, which means that a 50-Ω matched impedance is seen at the off-state rather than an open-circuit high impedance. Unlike the reflective switch, this absorptive switch minimizes the impedance mismatch and signal reflection at the off-state ports, and hence mitigates the phase and magnitude imbalances and radiation pattern degradations when the Butler matrix is connected to the switch. 

The switch is packaged in a land grid array (LGA) plastic package with 4 × 4 mm^2^ footprint. It is surface-mounted and soldered on the PCB. The switch has 24 pins, among which 4 pins are used for the four outputs, 1 pin is for the input, 2 pins are for the control voltages V_C1_, V_C2_, and the rest are for a positive supply VDD = +3.3 V, a negative supply VSS = −3.3 V, and ground GND = 0 V. V_C1_ and V_C2_ are set to either 0 V or +3.3 V for 2-bit control of the switching states. Five dc voltages of VDD, VSS, GND, V_C1_, V_C2_ are supplied through a 5-pin header placed at the lower side as illustrated by five solid dots in Figure 2. The width of the switch output pin is only 0.3 mm, which is narrower than the typical 50-Ω line width of 0.6 mm. Thus, the interconnection lines from the switch output pins to the Butler matrix input ports are designed in a stepped-impedance transformation configuration to minimize impedance mismatches and losses. Simulations have shown that the insertion loss due to the interconnection lines are made negligibly low, except for the switch’s inherent on-state insertion loss of 2.4 dB. 

Most part of the Butler matrix design is based on our prior design in [10]. In this design, the overall phase and amplitude imbalances of the Butler matrix are significantly improved by widening the bandwidth of the hybrid coupler. For the branch-line hybrid coupler, it is generally known that the multi-section structure can give wider bandwidth than the conventional single-section structure. We found that previous two-section [28,29] and three-section [30] designs demonstrated the bandwidth extension by 30–50%, but only in very low-RF band of 1–3 GHz. In contrast, in the mm-wave band, the conventional single-section design has been the most widely employed, for example, in Ka-band [10,11,12] and in V-band [13,15,21,23,24], which seems to be a limiting factor for the entire Butler matrix performances. Thus, in this design, we choose to adopt the two-section branch-line coupler design approach for our 28-GHz antenna. 

**Figure 3:**
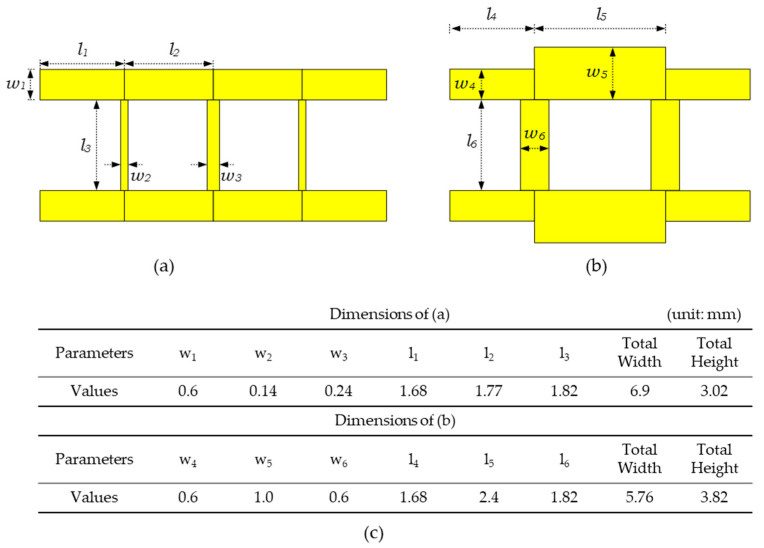
Brach-line hybrid coupler. (**a**) Proposed two-section structure, (**b**) conventional single-section structure, (**c**) dimensions.

**Figure 4:**
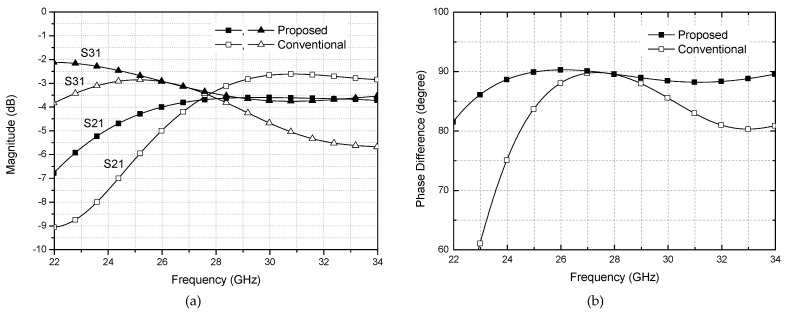
Simulation comparison of the branch-line couplers. (**a**) Magnitude imbalance, (**b**) phase imbalance.

Figure 3a shows the proposed two-section design, and Figure 3b shows the conventional single-section design of [10] for the purpose of comparison. The dimensions of the two designs are given in Figure 3c. It indicates that the proposed design does not necessarily occupy more area than the single-section one. A 5.3% area reduction is achieved in the proposed design. Figure 4 compares the EM simulation results for the two structures. It is clearly observed that the proposed two-section design gives wider bandwidth than the conventional design. For quantitative comparison, let us define the bandwidth as having <1 dB magnitude imbalance and <3° phase imbalance. Then, the operating bandwidth is dramatically extended from 26.8–28.6 GHz for the conventional design to 26–34 GHz for the proposed design, which is from 6.5% to 26.7% in term of the fractional bandwidth. We can also observe that the phase and amplitude imbalances over 26–30 GHz band are significantly improved from 4.5° to 1.5°, and from 2.1 dB to 1 dB, respectively.

**Figure 5:**
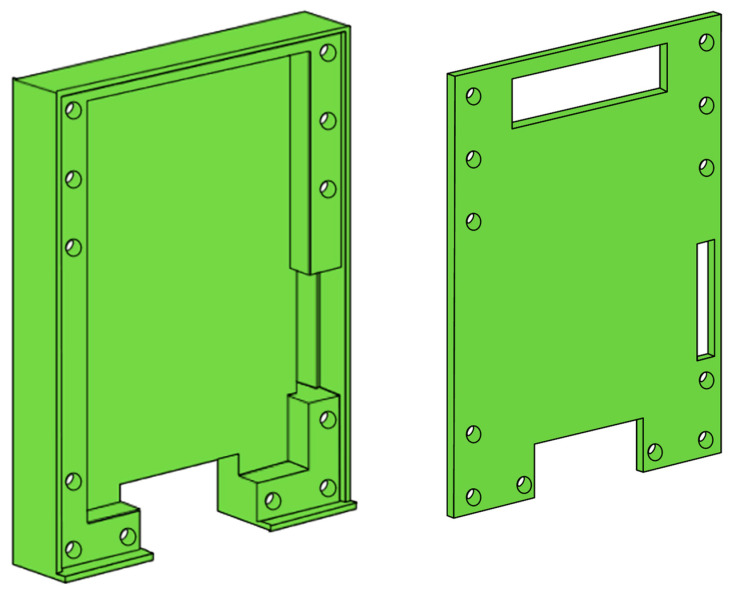
Metal enclosure.

Metal enclosure or housing for the entire antenna module improves the electrical performance as well as mechanical robustness. However, design of the metal enclosure and its co-integration with the antenna module were usually not described in the previous mm-wave switched-beam antennas [10,13,15,21,22,23,24]. Nevertheless, Ashraf et al. [12] claimed that a perfect magnetic conductor type enclosure could be effective for this purpose, but at the extra cost of sophisticated structure and fabrication process. Trinh-Van et al. [11] reported a perfect electric conductor type enclosure for their antenna, but no design details were described. In this work, we choose to design a perfect electric conductor type metal enclosure. Figure 5 shows the 3D view of the designed metal enclosure. It comprises a bottom box and top cover, which are to be assembled by 12 screws. The overall outer dimension of the enclosure is 37 × 50 × 6.2 mm^3^. Three openings are made at the top cover, which are the antenna array opening at the top, the RF connector opening at the bottom, and the dc pin header opening at the lower right side. Full 3D EM simulations verify that the antenna array opening area of 21.2 × 5.2 mm^2^ is wide enough not to affect the original radiation pattern when the enclosure is assembled. When the PCB of Figure 2 is put on the enclosure’s bottom box, a cavity is formed between the PCB back side and the enclosure’s bottom box. Then, the beamforming circuits on the PCB back side are exposed toward the cavity. If any cavity resonance occurs, it will change the impedances of the circuits and transmission lines, possibly leading to performance degradations [31]. Knowing the internal cavity dimension is 28 × 42.7 × 3 mm^3^, the dominant TE_001_ mode frequency is found to be 6.4 GHz. Through extensive EM simulations and verifications, we confirmed that the cavity resonances do not induce any significant performance degradations. 

All the design dimensions of the PCB of Figure 2 and metal enclosure of Figure 5 are carefully optimized through extensive full 3D EM simulations for the best performances. The EM simulation tool used in this work is the ANSYS high-frequency structure simulator (HFSS).

Table 1 summarizes the simulated performances of the Butler matrix at 28 GHz. By comparing the performances with and without the metal enclosure, we observe that the overall performance of the Butler matrix is not significantly affected by the metal enclosure. The performance parameters with the enclosure indicate that the maximum phase error is 7°, and the maximum extra loss (excluding the inherent 6-dB loss of an ideal Butler matrix) is +2.9 dB. These errors are found to impose negligible impacts on the final beam-switching performance and radiation patterns.

## 3. Results

**Figure 6:**
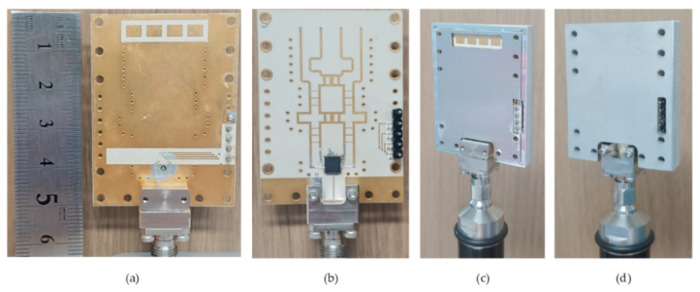
Photographs of the fabricated antenna. (**a**) Antenna side of PCB, (**b**) Butler matrix side of PCB, (**c**) antenna side of metal enclosure, (**d**) back side of metal enclosure.

The switched-beam antenna system is fabricated as shown in Figure 6. Figure 6a,b shows the PCB before the enclosure is assembled, and Figure 6c,d shows the antenna module after the enclosure assembled. The Taconic RF-30 substrate is used for the PCB. The RF-30 is made of a woven glass reinforced polytetrafluoroethylene (PTFE) material, and has a dielectric constant ε_r_ of 3.0, loss tangent tanδ of 0.0014 (measured at 1.9 GHz according to the product datasheet), and thickness h of 0.25 mm. The prepreg for attaching the two layers has a thickness of 0.1 mm and a dielectric constant ε_r_ of 4.0. Both sides of each layer are coated by copper with a thickness of 18 μm. The RF input signal is fed through a 2.92-mm RF end-launch connector assembled at the bottom side. The dc supply and control voltages are fed through the 5 pin headers located at the lower right side of the module. The LGA plastic package of the SP4T switch IC is located at the lower middle position in Figure 6b. The metal enclosure is made of aluminum. The overall module size with the enclosure closed is 37 × 50 × 6.2 mm^3^. 

**Figure 7:**
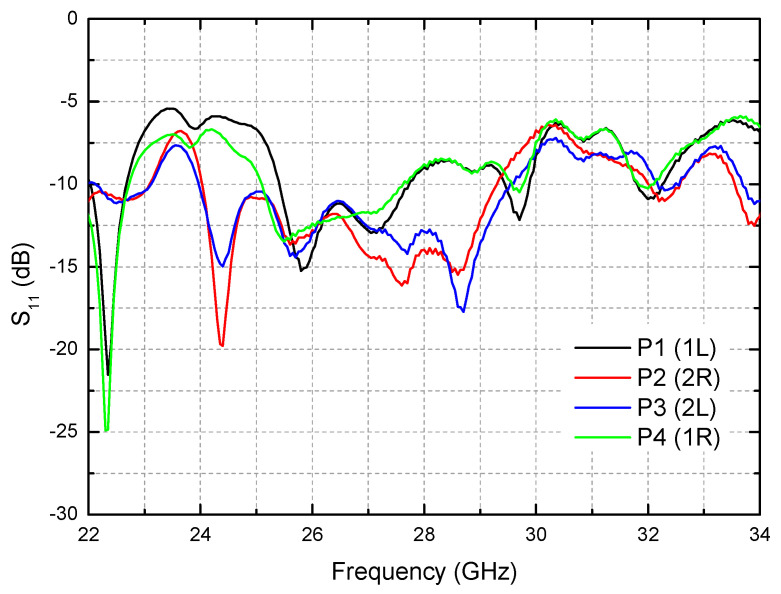
Measured S_11_.

The S-parameter of S_11_ at the RF input port was measured by using a vector network analyzer of Anritsu MS4647B. Figure 7 shows the measured S_11_ for the four switching states of P1–P4. The switching states are controlled by the 2-bit control voltages V_C1_, V_C2_ of either 0 or +3.3 V. At 28 GHz, S_11_ for the four switching states P1, P2, P3, P4 are −9, −14, −13, and −9 dB, respectively. It is found that S_11_ of P1 and P4 is slightly worse than that of P2 and P3. It can be accounted for by the fact that the routing lines from P1 and P4 to the Butler matrix are longer than the routing lines from P2 and P3, as can be observed in Figure 2b. Nevertheless, the four-state S_11_ are all found to be satisfactory. The bandwidth of S_11_ < −10 dB is 25.4–27.6 GHz. If a slightly relaxed bandwidth condition with S_11_ < −8 dB is adopted, the bandwidth becomes as wide as 25.2–29.6 GHz.

**Figure 8:**
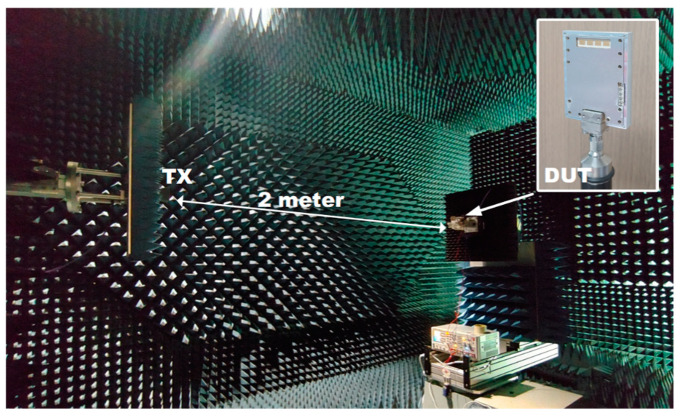
Anechoic chamber test setup for the radiation pattern over the air.

**Figure 9:**
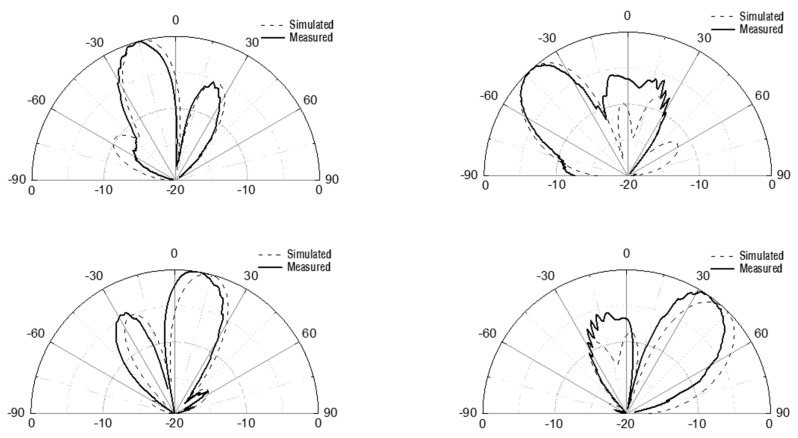
Measured and simulated radiation patterns of four directional beams in 28 GHz.

The radiation patterns are measured to verify the beam-switching operation of the fabricated antenna. Figure 8 is the over-the-air far-field measurement setup in an anechoic chamber. A reference horn antenna is located at the left side and the antenna under the test is located at the right side, and their distance is set to 2 m. The antenna under the test is characterized in a receiving mode. Figure 9 shows the measured results of the switched-beam radiation patterns in 28 GHz with comparison to the simulation results. Normalized gain is plotted for the sake of clear comparison. The beam steering angles are −43°, −17°, +10°, +34° for the measurements, while they are −44°, −16°, +18°, +44° for the simulations. The sidelobe levels are < −5.3 dB for the measurements, while they are < −6 dB for the simulations. The antenna gain is +5.8–+6.7 dBi for the four switched beams. Considering that our prior antenna of [10] has a gain of +8.5–+9.9 dBi, this gain values looks very reasonable because the additional insertion loss of 2.7–3.2 dB can be induced by the switch’s inherent insertion loss of 2.4 dB and its interconnecting lines with the Butler matrix and input RF-connector. Note that this antenna gain may be further increased by employing a series-fed structure like in [11]. Even though slight degradations of performances are observed throughout the measurements compared to the simulation results, overall performances of the fabricated antenna are found to be satisfactory. 

**Figure 10:**
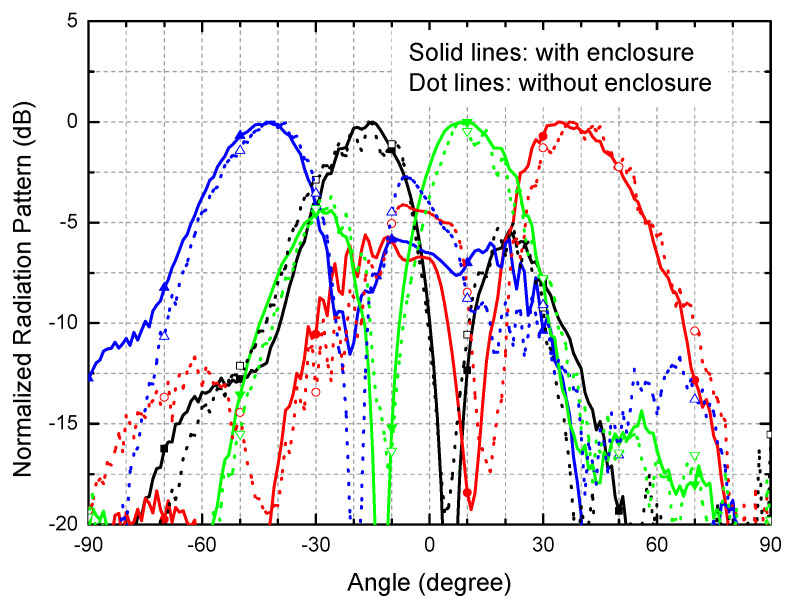
Measured radiation patterns with and without the metal enclosure in 28 GHz.

Figure 10 compares the 28-GHz radiation patterns with and without the metal enclosure. When the metal enclosure is assembled, the steering angles are slightly changed by 1–3°. The sidelobes are generally improved for all the four switched beams, and especially showed the biggest improvement from −2.7 dB to −5.8 dB for the 2L beam. In addition, the back lobes disappear almost perfectly. Thus, we conclude that the metal enclosure generally makes desirable effects and improvements on the radiation patterns. Such improvements are accounted for by the significant suppression of the parasitic radiations and unwanted electromagnetic couplings induced by the various circuit elements and interconnecting lines in the PCB.

**Figure 11:**
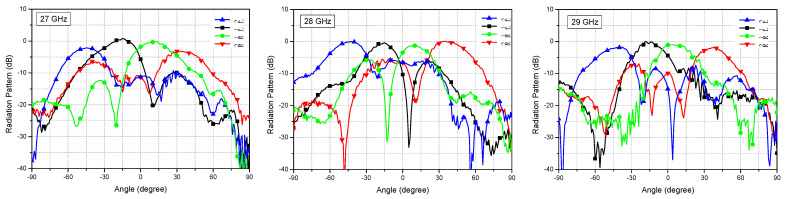
Measured radiation patterns in 27, 28, and 29 GHz.

Figure 11 shows the measured radiation patterns at three different frequencies of 27, 28 and 29 GHz. The four beam direction angles are found to be −45°/−43°/−41°, −15°/−17°/−17°, +13°/+10°/+8°, +34°/+34°/+38° at 27/28/29 GHz, respectively. Indeed, the beam direction angles changes slightly, but not significantly, with respect to the operating frequency. The peak gains of four beams are found to be constant at 28 and 29 GHz, showing less than 1 dB variation. However, at 27 GHz, the 2L/2R side-beams show a peak gain drop as high as 2.1–3.0 dB compared to the 1L/1R center-beams. The sidelobe levels are observed to be <3.1 dB at 27 GHz and <5.0 dB at 29 GHz, while it is <5.3 dB at 28 GHz. Overall, the antenna shows reasonably wideband performance over the frequency band of 27–29 GHz. When the operating frequency goes further out of the 27–29 GHz band, rapid performance degradations are also observed.

**Figure 12:**
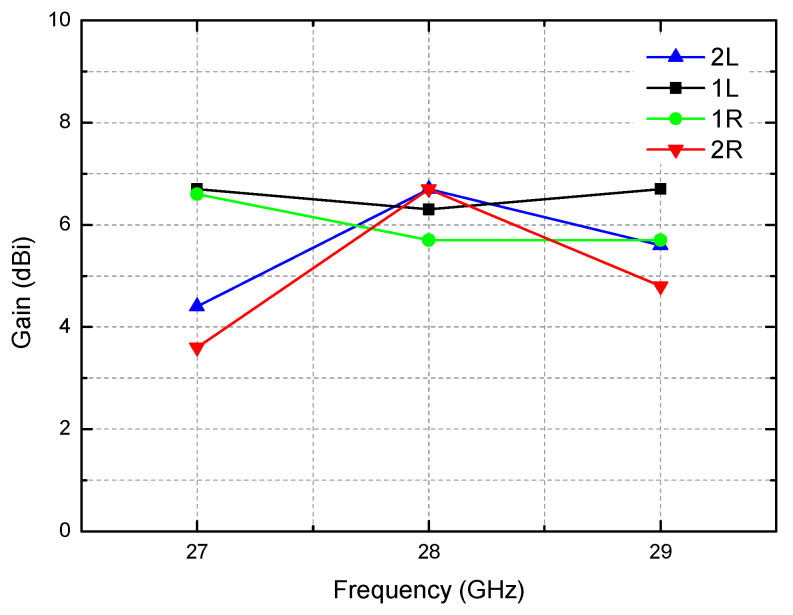
Antenna gains versus the frequency for the four directional beams.

The antenna gains for the four switched beams over the 27–29 GHz band are drawn in Figure 12. The antenna gains for the four beams are found to be +5.8–+6.7 dBi at 28 GHz. Since all the design optimization efforts have been focused on the 28-GHz center frequency performance, the four-beam antenna gains at 28 GHz show the least variation. However, the variation becomes greater, that is +3.6–+6.7 dBi at 27 GHz and +4.8–+6.7 dBi at 29 GHz. It is also interesting to note that the two side-beams of 2L/2R exhibit steeper gain drop than the center-beams of 1L/1R.

Table 2 summarizes and compares the performances of this work with the previous works. Since this work is focused on the full integration of all the key building blocks such as the antenna elements, Butler matrix, switch, and metal enclosure, the previous works [19,20,21,22,23,24] that also have demonstrated the similar integration level are chosen for comparison. However, their operating frequencies are not 28 GHz, but 24 GHz [21], 60 GHz [22,23,24], and 1.9–2.4 GHz [19,20]. Many 28-GHz switched-beam antennas can be found in the literature [3,4,5,6,7,8,9,10,11,12]. However, they all failed to achieve the full integration like this work, thus they may not be appropriate for this comparison. Nevertheless, [10,11,12] are included for comparison because they are based on the microstrip line structure like this work, while the others [3,4,5,6,7,8,9] are not included for comparison because they are based on non-microstrip line structure (the substrate integrated waveguide structure [3,4,5,6,7,8] and the substrate integrated suspended line structure [9]). Since no switch is integrated in [10,11,12], it should be noted that they must connect extra 50-Ω termination loads for the three unused Butler matrix ports during the test. The integrated switches in [21,22,23,24] include a specially fabricated MEMS switch [21], in-house designed proprietary CMOS switches [22,23], and commercially available but non-packaged bare-die switch [24]. Only this work adopts a commercially available off-the-shelf packaged switch chip. This will stabilize and improve the manufacturing process and cost. Meanwhile, in contrast to the reflective switches used in [21,24], the absorptive switch in this work as well as in [22,23] alleviate the possible impedance mismatch issue and thus improve the RF performances. The sidelobe level of this work is much better than [19,20,21,22] and comparable to [24]. However, it is found to be worse than [10,11,12] having no integrated switch. This work only utilizes a single SP4T switch like [21,22], whereas others utilize multiple SPDT switches [19,20,23,24]. 

For fair comparison of the total dimension, the normalized form factor as introduced in [10] is compared. It is the total area divided by the free-space wavelength squared and beam steering direction count. The normalized form factor of this work is 3.77, which is comparable to [10,21], and much better than [11,12,22,24]. The very low normalized form factor of [23] is achieved by employing an expensive multi-layer low temperature co-fired ceramic (LTCC) process rather than the low-cost conventional PCB process. In addition, the very small normalized form factors of [19,20] are accounted for by relaxed design allowances that can be depended on in the non-mm-wave low-GHz frequency range.

## 4. Conclusions

The 28-GHz Butler matrix based switched-beam antenna is successfully demonstrated for 5G wireless applications. It integrates a 1 × 4 microstrip antenna, a 4 × 4 Butler matrix, and absorptive SP4T packaged switch chip in a planar PCB and is housed in a metal enclosure. Design details of the co-integration of a packaged switch chip with the Butler matrix, the wideband two-section branch line coupler, and the metal enclosure are described for improving the RF performance and mechanical stability of the entire switched-beam antenna system. The fabricated antenna has a dimension of 37 × 50 × 6.2 mm^3^. The 10-dB impedance bandwidth is 25.4–27.6 GHz, while a slightly relaxed 8-dB bandwidth is 25.2–29.6 GHz. The antenna successfully demonstrates four directional switched beams with reasonably constant radiation patterns over the 27–29 GHz band. The advantages of high integration level and satisfactory RF performances of this work should be instrumental for the 28-GHz mm-wave 5G communication applications as well as other mm-wave wireless connectivity applications such as mm-wave radars and RF sensors.

## Figures and Tables

**Table 1:** Simulated performances of the Butler matrix.

	Port 1 (1L)			Port 2 (2R)			Port 3 (2L)			Port 4 (1R)		
Phase (Degree)	Without Enclosure	With Enclosure	Phase Error	Without Enclosure	With Enclosure	Phase Error	Without Enclosure	With Enclosure	Phase Error	Without Enclosure	With Enclosure	Phase Error
Port 5	45	45	0	135	135	0	90	90	0	180	180	0
Port 6	92	91	+1	5	2	+2	228	231	+6	134	128	−7
Port 7	133	129	−4	223	228	+3	10	7	+7	93	89	−1
Port 5	179	180	0	85	86	−4	140	133	−2	46	46	+1
**Insertion Loss (dB)**	**Without Enclosure**	**With Enclosure**	**Extra Loss**	**Without Enclosure**	**With Enclosure**	**Extra Loss**	**Without Enclosure**	**With Enclosure**	**Extra Loss**	**Without Enclosure**	**With Enclosure**	**Extra Loss**
Port 5	7.3	6.3	0.3	8.1	7.9	1.9	8.6	8.5	2.5	8.6	8.4	2.4
Port 6	9.3	8.3	2.3	10.1	8.9	2.9	7	6	0	9.8	8.6	2.6
Port 7	9.8	8.3	2.3	6.9	5.8	−0.2	10.2	9.5	3.5	9.3	8.5	2.5
Port 5	8.6	8.2	2.2	8.6	8.1	2.1	8.2	7.7	1.7	7.3	6.2	0.2

**Table 2:** Performance summary and comparison.

	Frequency (GHz)	Integrated Block Structure			Beam Direction Count	Maximum Beam Steering Angle (deg)	Sidelobe Level (dB)	Dimension (mm^2^)	Normalized Form Factor ^$^
		Antenna	Butler Matrix	Switch					
This work	28	1 × 4 Patch	4 × 4 Microstrip	Absorptive SP4T × 1 Commercial Silicon	4	−43, +34	−5.3	36 × 48	3.77
[10] Electr’19	28	1 × 4 Patch	4 × 4 Microstrip	No Switch	4	−39, +36	−6	36.2 × 44.3	3.48 ^&^
[11] TAP’19	28	4 × 7 Patch	4 × 4 Microstrip	No Switch	4	−39, +40	−9	95 × 32	6.63
[12] TMTT’21	30	1 × 4 Horn	4 × 4 Microstrip	No Switch	4	−42, +42	−7	50 × 52	6.50
[21] EuMIC’15	24	1 × 4 Patch	4 × 4 Microstrip	Reflective SP4T × 1 In-house MEMS ^§^	4	−55, +45	−1	74 × 33 ^†^	3.91
[24] TMTT’12	60	1 × 4 Dipole	4 × 4 Microstrip	Reflective SPDT × 3 Commercial GaAs PIN	4	−40, +40	−5	34.8 × 17.5 ^†^	6.09
[23] TMTT’12	63	1 × 4 Monopole	4 × 4 LTCC	Absorptive SPDT × 3 In-house CMOS	4	−48, +48	n/a	10 × 10	1.0
[22] TMTT’10	60	1 × 4 Patch	4 × 4 CMOS	Absorptive SP4T × 1 In-house CMOS	4	−38, +38	−2	45 × 55	24.7
[19] TMTT’17	2.4	4 × 4 Patch	4 × 4 Microstrip	Reflective SPDT × 2 ^§§^ In-house GaAs	16 (4 H × 4 V)	−49, +49	−3	320 × 312	0.4
[20] Electr’19	1.96	1 × 4 Slot Patch	4 × 4 Microstrip	Absorptive SP4T × 1, SPDT × 4 Commercial	4	−39, +31	−2	342 × 87 + 110 × 110 ^†^	0.47

^&^ Calculated for the whole structure dimension including the surrounding ground plane. ^§^ MEMS switch requires a driving voltage as high as 50 V. ^§§^ Only two SPDT is used because an off-state is realized in the SPDT. ^†^ Estimated from the reported photos. ^$^ Calculated by total area/(λ^2^ × beam direction count), in which λ is the free-space wavelength.
